# Uniportal Robotic-Assisted Versus Video-Assisted Thoracoscopic Surgery for Anatomical Lung Resection in Non-Small Cell Lung Cancer: A Comparative Single-Center Cohort Study

**DOI:** 10.3390/jcm15114078

**Published:** 2026-05-25

**Authors:** Mehlika İşcan, Ömer Yavuz, Reyhan Ertan, Ali Yeginsu

**Affiliations:** Department of Thoracic Surgery, Başakşehir Çam ve Sakura City Hospital, 34480 Istanbul, Türkiyerertanertan@gmail.com (R.E.); yeginsu@gmail.com (A.Y.)

**Keywords:** uniportal robotic surgery, uniportal VATS, non-small cell lung cancer, anatomical lung resection, lymph node dissection, textbook outcome, minimally invasive thoracic surgery

## Abstract

**Background:** Direct comparisons between uniportal robotic-assisted (uRATS) and uniportal video-assisted (uVATS) thoracoscopic anatomical lung resection for non-small cell lung cancer (NSCLC) remain scarce. We compared oncologic radicality and perioperative outcomes between the two uniportal approaches in a single-center contemporaneous cohort. **Methods:** This retrospective cohort study included 56 consecutive NSCLC patients undergoing uniportal anatomical resection between January 2024 and December 2025 (uRATS, *n* = 12; uVATS, *n* = 44). The primary endpoint was oncologic radicality of lymph-node dissection (stations sampled, total nodes, mediastinal sampling, R0 rate). Secondary endpoints included operative time, blood loss, pain, recovery metrics, and a composite textbook outcome. Comparisons used Mann–Whitney U and Fisher’s exact tests. **Results:** Complete (R0) resection was achieved in all 56 patients. The operating surgeon dissected more lymph nodes in the uRATS group (median 13 vs. 7; *p* = 0.049), with a trend toward more mediastinal stations sampled (4 vs. 3; *p* = 0.061). Operative time was longer with uRATS (220 vs. 135 min; *p* < 0.001), but air-leak duration (0 vs. 2 days; *p* < 0.001), hospital stay (2 vs. 3 days; *p* = 0.022), and discharge pain (*p* = 0.017) all favored uRATS. Textbook outcome was achieved in 83% versus 48% (*p* = 0.047). **Conclusions:** In a uniportal-experienced unit, uRATS showed comparable intraoperative oncologic-quality metrics to uVATS with directional perioperative-recovery differences favoring uRATS. Larger multicenter studies with longer follow-up are warranted.

## 1. Introduction

Lung cancer remains the most commonly diagnosed malignancy and the leading cause of cancer-related death worldwide, accounting for approximately 2.5 million new cases (12.4% of all incident cancers) and 1.8 million deaths (18.7% of all cancer deaths) in 2022 [1]. Non-small cell lung cancer (NSCLC) represents roughly 85% of all lung cancer cases, and for patients with resectable disease, anatomical pulmonary resection with systematic mediastinal lymph-node dissection remains the cornerstone of curative-intent treatment [2,3]. The extent of intraoperative lymph-node evaluation has consistently been identified as an independent determinant of accurate pathological staging and long-term oncologic outcome, and the European Society of Thoracic Surgeons (ESTS) recommends sampling of at least three mediastinal stations, including the subcarinal station 7, as a minimum standard of surgical quality [4].

Over the past three decades, surgical management of early-stage NSCLC has progressively shifted from open thoracotomy to minimally invasive approaches, driven by growing evidence that video-assisted thoracoscopic surgery (VATS) lobectomy offers equivalent oncologic efficacy to open resection while reducing perioperative morbidity, postoperative pain, and hospital stay [5,6]. The uniportal VATS (uVATS) technique, first described for anatomical lobectomy by Gonzalez-Rivas et al. in 2011, has further refined this minimally invasive paradigm by delivering all instruments and the thoracoscope through a single intercostal incision without rib spreading, thereby minimizing chest-wall trauma and potentially improving pain control and early recovery [7,8]. Meta-analytic evidence comparing uVATS with multiportal VATS has reported comparable oncologic outcomes, R0 resection rates, and lymph-node retrieval, with a trend toward reduced postoperative pain in uVATS [9,10].

In parallel with the evolution of uniportal thoracoscopy, robotic-assisted thoracic surgery (RATS) has emerged as a technological advance that couples three-dimensional stereoscopic visualization, tremor filtration, and wristed instrument articulation, offering potential advantages for the precise dissection of hilar and mediastinal structures [11]. Contemporary systematic reviews and meta-analyses—including randomized trial evidence-have demonstrated that multiportal RATS and VATS lobectomy yield equivalent long-term overall and disease-free survival in NSCLC, with RATS showing advantages in the number of lymph nodes dissected and length of hospital stay in several analyses, while perioperative complications, conversion rates, and prolonged air leak are broadly comparable [12,13,14]. A 2023 reconstructed-patient-data meta-analysis with a median follow-up of 51.7 months confirmed equivalence of overall and disease-free survival between the two approaches while demonstrating shorter hospital stay and higher lymph-node yield with RATS [12].

The logical convergence of the uniportal philosophy with robotic technology has given rise to uniportal robotic-assisted thoracic surgery (uRATS), first performed for anatomical lobectomy in 2021 using an adapted da Vinci Xi platform [15]. Initial European multicenter experiences and single-surgeon propensity-matched analyses have reported the safety and feasibility of uRATS for pulmonary anatomical resection, with acceptable conversion rates, low perioperative morbidity, and short hospital stay [16,17]. Subsequent cohorts have further refined the technical approach, including modifications to incision length, trocar configuration, and the use of a three-arm (camera plus two operating arms) setup suitable for the intercostal approach [18,19]. Dedicated single-port robotic platforms and subcostal approaches have also been investigated in pilot trials, expanding the armamentarium of minimally invasive strategies [20,21]. Nevertheless, direct comparisons between uRATS and uVATS for anatomical resection of NSCLC—the two most minimally invasive options available to the uniportal-trained surgeon—remain scarce, and the majority of available evidence to date focuses on comparisons of uRATS with multiportal RATS rather than with its uniportal thoracoscopic predecessor [16,22].

This evidentiary gap is of particular relevance to contemporary thoracic surgery practice, because the clinically meaningful question for a unit already proficient in uniportal thoracoscopy is not whether robotics adds value over multi-portal approaches, but whether the transition from uVATS to uRATS delivers incremental benefit—in oncologic radicality, perioperative recovery, or both—sufficient to justify the additional resources, learning curve, and operating time associated with the robotic platform. Comprehensive comparisons that simultaneously evaluate oncologic endpoints (lymph-node yield, station sampling, R0 resection) and perioperative recovery endpoints (operative time, blood loss, pain, air-leak duration, hospital stay, textbook outcome) within a single institution and a contemporaneous time frame are therefore needed to inform this practical question.

The aim of the present study was to compare oncologic radicality and perioperative outcomes between uRATS and uVATS anatomical lung resection for NSCLC in a single-center cohort. The primary endpoint was oncologic radicality of lymph-node dissection, assessed through total lymph-node stations sampled, total lymph-node yield, mediastinal (N2) station sampling, and R0 resection rate. Secondary endpoints included operative and docking/console times, estimated blood loss, postoperative pain, duration of air leak, chest-tube and hospital stay, 30-day morbidity and mortality, and a composite “textbook outcome” adapted from contemporary thoracic-surgery literature [23]. We hypothesized that, in the hands of a surgical team experienced in the uniportal thoracoscopic approach, uRATS would provide comparable short-term oncologic-quality metrics to uVATS, with potential directional differences in perioperative recovery.

## 2. Materials and Methods

### 2.1. Study Design and Setting

This was a single-center, retrospective observational cohort study conducted at the Department of Thoracic Surgery of our tertiary referral hospital. The study compared all consecutive patients who underwent uRATS with those who underwent uVATS for anatomical pulmonary resection of non-small cell lung cancer (NSCLC) during a contemporaneous period. The analysis plan and outcome definitions were specified before data extraction, and the study was reported in accordance with the Strengthening the Reporting of Observational Studies in Epidemiology (STROBE) statement for cohort studies. The study period spanned from January 2024 through December 2025.

### 2.2. Ethical Approval and Consent

The study was conducted in accordance with the Declaration of Helsinki and was approved by the Başakşehir Çam ve Sakura Şehir Hastanesi Bilimsel Araştırmalar 1 No'lu Etik Kurulu (Approval No: KAEK/24.12.2025.396; Approval Date: 24 December 2025). Written informed consent for the surgical procedure and the subsequent use of de-identified clinical data for research purposes was obtained from all participants prior to the intervention.

### 2.3. Patient Selection

All consecutive patients aged ≥18 years who underwent uniportal minimally invasive anatomical lung resection (segmentectomy, lobectomy, or pneumonectomy) for histologically confirmed NSCLC during the study period were screened for inclusion. Patients undergoing non-anatomical wedge resection only, resection for non-NSCLC pathology (including pulmonary metastasectomy, benign disease, and hydatid cyst), as well as resections performed through multi-portal or open approaches, were excluded.

A uniportal minimally invasive approach was offered to patients with clinical T1–T4 NSCLC without chest-wall, mediastinal, or great-vessel invasion, who had adequate cardiopulmonary reserve to tolerate single-lung ventilation. Once a patient was deemed suitable for a uniportal anatomical resection, the choice between uRATS and uVATS was governed by the day-to-day availability of the robotic platform, which was a shared institutional resource and was not available on every operative day; on days when the robotic system was not available, eligible patients underwent uVATS by the same operating surgical team. No patient-level clinical criterion—including predicted FEV_1_ percentage, comorbidity burden, age, body-mass index, The American Society of Anesthesiologists (ASA) physical status and the Eastern Cooperative Oncology Group (ECOG) performance status, tumor size or clinical stage, or anatomical lobe—was used to assign patients between the two approaches. Informed consent for both potential approaches was obtained from each patient prior to scheduling. This platform-availability-driven allocation, conceptually analogous to the framework previously employed by Huang and colleagues in a comparative cohort of robotic and thoracoscopic anatomical lung resection [24], was intended to minimize patient-level selection bias. Given the small size of the uRATS group and the constrained number of covariates, formal propensity-score matching was considered but not performed.

Despite this allocation strategy, baseline pulmonary-function values were modestly higher in the uRATS group than in the uVATS group, as detailed in Section 3.1 (Table 1). This residual imbalance is acknowledged as an unintended consequence of non-randomized assignment rather than a reflection of any deliberate clinical-selection algorithm; its potential implications for outcome interpretation are addressed in Section 5 (Discussion, Limitations).

### 2.4. Preoperative Evaluation and Clinical Staging

All patients underwent a standardized preoperative assessment, including a complete medical history, physical examination, resting 12-lead electrocardiography, complete blood count, comprehensive metabolic panel, and pulmonary function tests (spirometry with forced expiratory volume in one second (FEV_1_) expressed as both absolute value in liters and percentage of predicted, and FEV_1_/FVC ratio). Transthoracic echocardiography with left ventricular ejection fraction (LVEF) was performed in patients with cardiovascular risk factors. The ASA physical status and the ECOG performance status were recorded preoperatively. The age-adjusted Charlson Comorbidity Index (aCCI) was calculated from the documented comorbidities, with an additional 1-point score assigned for any chronic pulmonary disease (COPD, asthma, interstitial lung disease, or bronchiectasis), and the diagnosis of primary lung cancer was added to the CCI weight. Clinical staging was based on contrast-enhanced thoracic computed tomography (CT), whole-body [^18^F]-fluorodeoxyglucose positron emission tomography/CT (PET/CT), and contrast-enhanced cranial magnetic resonance imaging (MRI). Tumor size was recorded on both CT and PET/CT in millimeters (mm). Mediastinal staging was confirmed by EBUS-TBNA and/or videomediastinoscopy in patients with PET/CT-positive mediastinal lymph nodes, suspected N1 disease, central tumors, or tumors > 3 cm, in accordance with current ESTS recommendations. Clinical TNM staging followed the 9th edition of the UICC/AJCC TNM classification for lung cancer.

### 2.5. Surgical Procedures

#### 2.5.1. General Principles

All procedures were performed under general anesthesia with double-lumen endotracheal intubation and selective one-lung ventilation, with the patient in the lateral decubitus position. Preoperative antibiotic prophylaxis and intraoperative warming were standard. All uRATS procedures were performed by a single thoracic surgeon experienced in multiportal robotic-assisted, uniportal video-assisted thoracoscopic, and uniportal robotic-assisted approaches. All uVATS procedures were performed by a small team of thoracic surgeons that included the same surgeon who performed all uRATS cases, thereby ensuring continuity of surgical philosophy and oncological resection principles across both arms.

#### 2.5.2. Uniportal VATS (uVATS) Technique

A single 3–4 cm incision was placed in the fifth intercostal space at the anterior axillary line, without rib spreading. An endoscopic wound-protector retractor was placed at the incision to facilitate instrument exchange and protect the wound edges. The camera and all working instruments were introduced through this single incision. Anatomical dissection of the hilar vessels and bronchus was carried out using endoscopic staplers, energy devices, and conventional thoracoscopic instruments. Systematic mediastinal lymph-node dissection was performed in all cases in accordance with the ESTS guideline.

#### 2.5.3. Uniportal Robotic Assisted (uRATS) Technique

All uRATS procedures were performed using the da Vinci Xi surgical system (Intuitive Surgical, Sunnyvale, CA, USA) through a single 4 cm incision placed in the fifth intercostal space at the anterior axillary line. An Alexis endoscopic wound-protector retractor (Applied Medical, Rancho Santa Margarita, CA, USA) was placed at the incision, through which the 8 mm robotic instrument cannulas and the robotic camera were introduced. Three robotic arms were employed in all cases—the camera arm, a Cadiere forceps, and a Maryland bipolar forceps; the fourth robotic arm was not used. CO_2_ insufflation was not employed; selective one-lung ventilation provided adequate pleural exposure. Anatomical dissection proceeded in a fashion identical to the uVATS technique. Vascular and bronchial transections were performed with manual (non-robotic) endoscopic staplers introduced through the uniportal incision by the bedside assistant. Docking time was defined as the interval from positioning of the cart to completion of instrument calibration; console time was defined as the duration of active robotic dissection. Systematic mediastinal lymph-node dissection was performed in all cases.

### 2.6. Postoperative Management

According to our institutional protocol, postoperative transfer to the intensive care unit (ICU) for overnight monitoring is the standard of care following anatomical lung resection and does not reflect a clinical complication. Both routine overnight ICU monitoring and direct postoperative transfer to the surgical ward are institutionally accepted pathways. Most patients in both groups followed the routine ICU pathway; the remainder were transferred directly to the ward. The median ICU length of stay among admitted patients was 12 h, consistent with routine overnight observation. A single 28 Fr chest drain was placed at the end of each operation and managed with underwater seal drainage. Chest-tube removal was performed when there was no air leak, when pleural fluid output was <200 mL per 24 h, and when the post-expiratory chest radiograph showed full lung re-expansion. Postoperative pain was managed with a multimodal regimen (intercostal block, paracetamol, a non-steroidal anti-inflammatory agent, and opioid rescue as required).

### 2.7. Outcome Measures and Definitions

#### 2.7.1. Primary Endpoint

The primary endpoint was the oncologic radicality of intraoperative lymphadenectomy. To meaningfully separate the surgical component of nodal yield from the pathology-dependent component, our institutional protocol uses an explicit two-compartment specimen-handling and reporting strategy, in line with contemporary station-based quality standards for lung cancer surgery.

Mediastinal stations (e.g., stations 2, 4, 7, 8, and 9 on the right side; stations 5, 6, 7, 8, and 9 on the left side) and the hilar station 10 were dissected intraoperatively and submitted to pathology in separate, station-labeled containers. Station 11 was either dissected intraoperatively by the operating surgeon and submitted in a separate container or retrieved from within the en bloc lobectomy specimen by the pathologist, depending on intraoperative anatomy and exposure. The combined number of nodes from all stations submitted separately by the operating surgeon is hereafter referred to as the “surgeon-dissected lymph-node count” and reflects the intraoperative completeness of the mediastinal and extralobar lymphadenectomy. The remaining intralobar N1 stations (i.e., stations 12, 13, and 14, and station 11 when not separately submitted) were left within the en bloc lobectomy specimen and subsequently retrieved by the reviewing pathologist during gross specimen examination; this is hereafter referred to as the “specimen-retrieved lymph-node count” and depends largely on the thoroughness of pathological processing. The total lymph-node yield is the sum of these two compartments.

The primary endpoint comprised the following pre-specified metrics: (i) the total number of mediastinal lymph-node stations sampled per patient; (ii) the proportion of patients in whom at least three mediastinal stations including station 7 were sampled, in accordance with the ESTS recommendation; (iii) the surgeon-dissected lymph-node count, selected as the primary nodal-yield variable because it is anatomically and operationally distinct from pathologist-dependent N1 retrieval; (iv) the total lymph-node yield (surgeon-dissected plus specimen-retrieved); (v) the proportion of patients with adequate yield (≥10 lymph nodes); and (vi) the rate of complete (R0) resection [4,25].

#### 2.7.2. Secondary Endpoints

Secondary endpoints included operative time (minutes), docking time and console time (minutes, uRATS only), estimated intraoperative blood loss (mL), intraoperative transfusion requirement, conversion to an alternative surgical approach, technical difficulty, postoperative pain measured by the 10-point Visual Analog Scale (VAS) at 12 h postoperatively (VAS_12_h) and at discharge (VAS_discharge), duration of air leak (days), prolonged air leak defined as >5 days, chest-tube duration (days), hospital stay (days), any in-hospital complication, minor complication (Clavien–Dindo grade I–II), major complication (Clavien–Dindo grade ≥ III), 30-day readmission, and 30-day mortality. A composite “textbook outcome” was defined, adapting published definitions for thoracic surgery, as the simultaneous achievement of all of the following: R0 resection, absence of major (Clavien ≥ III) complication, absence of prolonged air leak, no 30-day readmission, no 30-day mortality, and ≥6 lymph-node stations sampled [23]. Routine overnight ICU admission was not included in the textbook-outcome composite because it reflects institutional standard-of-care rather than a clinical event.

#### 2.7.3. Pathological Outcomes

Histological classification followed the 2021 World Health Organization (WHO) classification of thoracic tumors [26]. Pathological TNM staging was assigned according to the 9th edition of the TNM classification for lung cancer proposed by the International Association for the Study of Lung Cancer (IASLC) and subsequently adopted by the UICC/AJCC [27]. Tumor size (longest diameter of the pathological specimen) was recorded in centimeters (cm). Surgical margin distance was recorded in centimeters. Complete (R0) resection was defined as microscopically negative resection margins on histopathological examination, in accordance with the IASLC criteria [28]. The reviewing pathologist was aware that the specimen represented an anatomical lung resection with mediastinal lymph-node dissection, but was not informed of the specific surgical approach used (uRATS vs. uVATS) at the time of specimen examination, and the pathology request form did not specify the surgical platform. The presence of lymphatic, vascular, and perineural invasion, as well as visceral pleural invasion, was recorded as dichotomous variables. Nodal upstaging was defined as a change from clinical N0 status preoperatively to pathological N1 or N2 status postoperatively.

### 2.8. Statistical Analysis

Data are reported as median with interquartile range (IQR, represented as 25th to 75th percentiles) for continuous variables and as frequencies with percentages for categorical variables. Non-parametric descriptive statistics were selected a priori owing to the small size of the uRATS group (*n* = 12) and the non-normal distribution of several outcome variables. Between-group comparisons of continuous variables were performed using the Mann–Whitney U test. Categorical variables were compared using Fisher’s exact test, which was preferred over the chi-square test given the small sample sizes encountered in this cohort. Ordered categorical variables (e.g., pathological T stage, pathological stage) were analyzed using Fisher’s exact test with Monte Carlo simulation.

For the primary endpoint (lymph-node yield and station sampling), a two-tailed α of 0.05 was considered statistically significant. No formal adjustment for multiple comparisons was applied, and all reported *p*-values are unadjusted.

Baseline comparability of the two groups was assessed without formal propensity-score matching, given the sample-size imbalance (12 vs. 44) that would render matched subsets uninformative. The age-adjusted Charlson Comorbidity Index, ASA physical status, and ECOG performance status were each compared between groups to provide a comprehensive description of preoperative risk. Missing data were handled by complete-case analysis; the absolute number of missing observations for each variable is reported where applicable. No imputation was performed, given the low overall rate of missingness (<3% for primary endpoint variables and <5% for all variables retained in the analysis).

Figures were generated using Python (version 3.12) with plot types appropriate to the underlying data structure: violin plots with individual-patient swarm overlay for the primary endpoint (lymph-node yield), box-and-whisker plots with swarm overlay for continuous perioperative variables, a STROBE flow diagram for patient selection, and a grouped bar chart for dichotomous outcome comparisons. All statistical computations were performed using Python (version 3.12) with the SciPy (version 1.11), statsmodels (version 0.14), and pandas (version 2.1) libraries. All reported *p*-values are two-tailed. A *p*-value < 0.05 was considered statistically significant.

## 3. Results

### 3.1. Cohort and Baseline Characteristics

**Figure 1:**
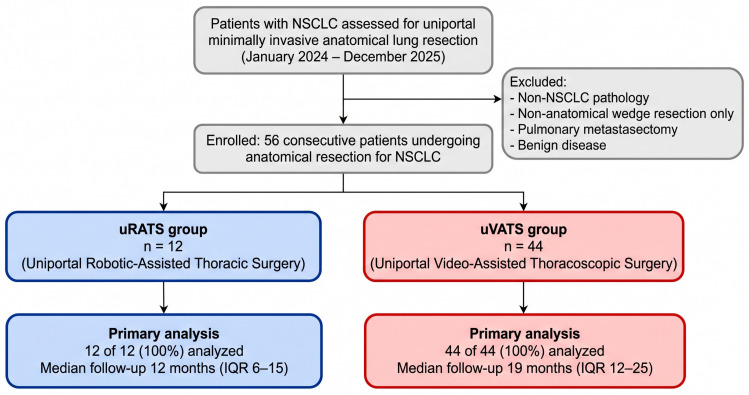
STROBE flow diagram of patient selection and study cohort assembly. During the study period (January 2024 through December 2025), all consecutive patients with non-small cell lung cancer (NSCLC) assessed for uniportal minimally invasive anatomical lung resection were screened. Patients with non-NSCLC pathology, non-anatomical wedge resection only, pulmonary metastasectomy, or benign pulmonary disease were excluded. The final cohort comprised 56 patients who underwent anatomical resection for NSCLC: 12 received uniportal robotic-assisted thoracic surgery (uRATS) and 44 received uniportal video-assisted thoracoscopic surgery (uVATS). All patients were included in the primary analysis. Median follow-up was 12 months (interquartile range 6–15) for the uRATS group and 19 months (interquartile range 12–25) for the uVATS group. Abbreviations: NSCLC, non-small cell lung cancer; uRATS, uniportal robotic-assisted thoracic surgery; uVATS, uniportal video-assisted thoracoscopic surgery; IQR, interquartile range; STROBE, strengthening the reporting of observational studies in epidemiology.

During the study period (January 2024 through December 2025), 56 consecutive patients underwent uniportal minimally invasive anatomical lung resection for histologically confirmed NSCLC and were included in the analysis; 12 patients (21.4%) received uRATS, and 44 patients (78.6%) received uVATS (Figure 1). No patient was lost to follow-up, and all were analyzed in their assigned group (complete-case follow-up, 100% in both arms). Median follow-up was 12 months (IQR 6–15) in the uRATS group and 19 months (IQR 12–25) in the uVATS group.

Baseline demographic, functional, and oncologic characteristics are summarized in Table 1. The two groups were comparable in age (median 67 years [IQR 61–74] vs. 68 years [IQR 62–72]; *p* = 0.881), sex distribution (male/female 8/4 vs. 34/10; *p* = 0.470), body mass index (27.6 kg/m^2^ [IQR 22.8–29.4] vs. 27.0 kg/m^2^ [IQR 25.0–30.0]; *p* = 0.749), ever-smoker status (83.3% vs. 84.1%; *p* = 1.000), and cumulative tobacco exposure (median 45 pack-years in both groups; *p* = 1.000). No significant between-group differences were observed in the Charlson Comorbidity Index (median 5 vs. 4; *p* = 0.264), age-adjusted CCI (median 7 vs. 6; *p* = 0.207), proportion of patients with ASA physical status ≥ III (58.3% vs. 61.4%; *p* = 1.000), or ECOG performance status ≥ 1 (58.3% vs. 68.2%; *p* = 0.516). Individual comorbidities—chronic obstructive pulmonary disease, diabetes mellitus, hypertension, and ischemic heart disease—were similarly distributed between groups (all *p* > 0.05). The prevalence of previous or concurrent other malignancy was also comparable (25.0% vs. 18.2%; *p* = 0.686). Clinical tumor size on contrast-enhanced thoracic CT (median 27 mm vs. 24 mm; *p* = 0.944) and on PET/CT (24 mm vs. 24 mm; *p* = 0.928), as well as tumor metabolic activity (SUVmax 7.0 vs. 8.6; *p* = 0.920), did not differ between groups. Neoadjuvant oncologic treatment for lung cancer had been administered to one patient in the uVATS group (2.3%) and to no patient in the uRATS group (*p* = 1.000).

Two differences reached statistical significance in preoperative pulmonary function: the uRATS group exhibited higher predicted FEV_1_ percentage (median 102% [IQR 91–106] vs. 85% [IQR 75–102]; *p* = 0.029) and a higher FEV_1_/FVC ratio (106% [IQR 104–114] vs. 99% [IQR 92–106]; *p* = 0.048). These differences were modest in absolute terms, and all uVATS patients nevertheless met institutional criteria for anatomical resection; the implications for outcome interpretation are addressed in the Discussion.

### 3.2. Intraoperative and Surgical Characteristics

**Figure 2:**
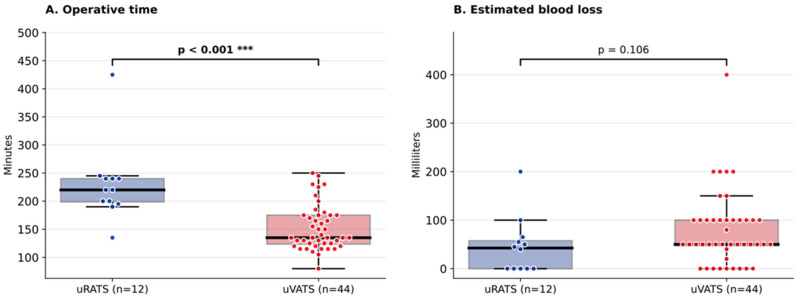
Intraoperative parameters. Comparison of two intraoperative variables between the uRATS (blue; *n* = 12) and uVATS (red; *n* = 44) groups. (**A**) Total operative time in minutes, measured from skin incision to wound closure. (**B**) Estimated intraoperative blood loss in milliliters. Box plots display the median (central horizontal line) and the interquartile range (box margins); whiskers extend to the most extreme data point within 1.5 × IQR of the nearest quartile, and individual dots represent each patient. Between-group comparisons were performed using the Mann–Whitney U test; *** *p* < 0.001. Abbreviations: IQR, interquartile range; uRATS, uniportal robotic-assisted thoracic surgery; uVATS, uniportal video-assisted thoracoscopic surgery.

Intraoperative details are presented in Table 2. The distribution of resection types was comparable between groups (lobectomy 66.7% vs. 77.3%; segmentectomy 25.0% vs. 20.5%; pneumonectomy 8.3% vs. 2.3%; overall *p* = 0.548). Operative side (right/left: 9/3 vs. 26/18; *p* = 0.501) and lobe/segment location did not differ. In the uRATS group, median docking time was 5 min (IQR 5–5) and median console time was 190 min (IQR 179–205). The total operative time was, as anticipated, significantly longer in the uRATS group (median 220 min [IQR 199–240]) than in the uVATS group (135 min [IQR 124–175]; *p* < 0.001; Figure 2A). Estimated intraoperative blood loss showed a numerical trend toward lower values in the uRATS group (42 mL [IQR 0–58] vs. 50 mL [IQR 48–100]; *p* = 0.106; Figure 2B), but this difference did not reach statistical significance. No intraoperative red-cell transfusion and no conversion to multi-portal or open surgery occurred in either group. One minor intraoperative event was documented in the uVATS group (2.3%) and none in the uRATS group (*p* = 1.000).

### 3.3. Postoperative Outcomes, Pain, and Recovery

**Figure 3:**
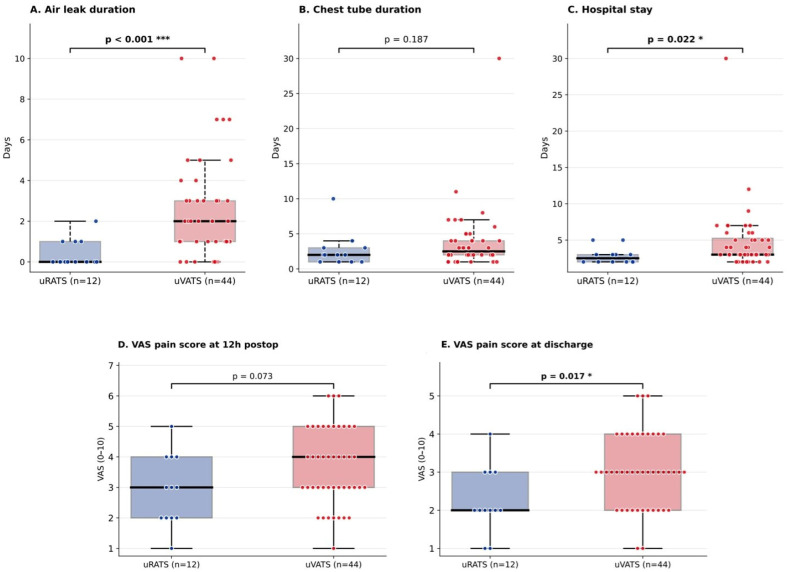
Postoperative pain and recovery. Comparison of five postoperative endpoints between the uRATS (blue; *n* = 12) and uVATS (red; *n* = 44) groups. (**A**) Duration of postoperative air leak in days. (**B**) Chest-tube duration in days. (**C**) Total hospital length of stay in days. (**D**) Postoperative pain was measured on a 10-point Visual Analog Scale (VAS) at 12 h following surgery. (**E**) VAS pain score at hospital discharge. Box plots display the median (central horizontal line) and the interquartile range (box margins); whiskers extend to the most extreme data point within 1.5 × IQR of the nearest quartile, and individual dots represent each patient. Between-group comparisons were performed using the Mann–Whitney U test; * *p* < 0.05, *** *p* < 0.001. Abbreviations: IQR, interquartile range; VAS, Visual Analog Scale; uRATS, uniportal robotic-assisted thoracic surgery; uVATS, uniportal video-assisted thoracoscopic surgery.

**Figure 4:**
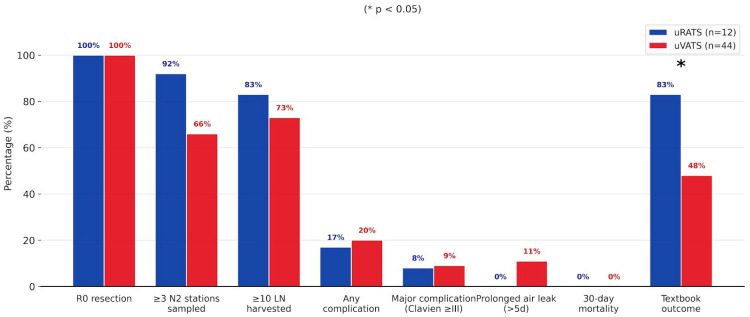
Dichotomous oncologic and perioperative outcomes. Grouped bar chart comparing the proportion (%) of patients achieving each binary outcome in the uRATS (blue; *n* = 12) and uVATS (red; *n* = 44) groups. Outcomes shown, from left to right: complete (R0) resection; sampling of ≥3 mediastinal (N2) stations, corresponding to the European Society of Thoracic Surgeons (ESTS) lymphadenectomy quality criterion; adequate lymph-node yield (≥10 nodes harvested); any in-hospital complication; major complication (Clavien–Dindo grade ≥ III); prolonged air leak (>5 days); 30-day all-cause mortality; and textbook outcome (a composite metric defined as the simultaneous achievement of R0 resection, absence of major complication, absence of prolonged air leak, no 30-day readmission, no 30-day mortality, and sampling of ≥6 lymph-node stations). Percentage values are displayed above each bar. Between-group comparisons were performed using Fisher’s exact test; * *p* < 0.05. Abbreviations: LN, lymph node; N2, mediastinal nodal station; ESTS, European Society of Thoracic Surgeons; uRATS, uniportal robotic-assisted thoracic surgery; uVATS, uniportal video-assisted thoracoscopic surgery.

**Figure 5:**
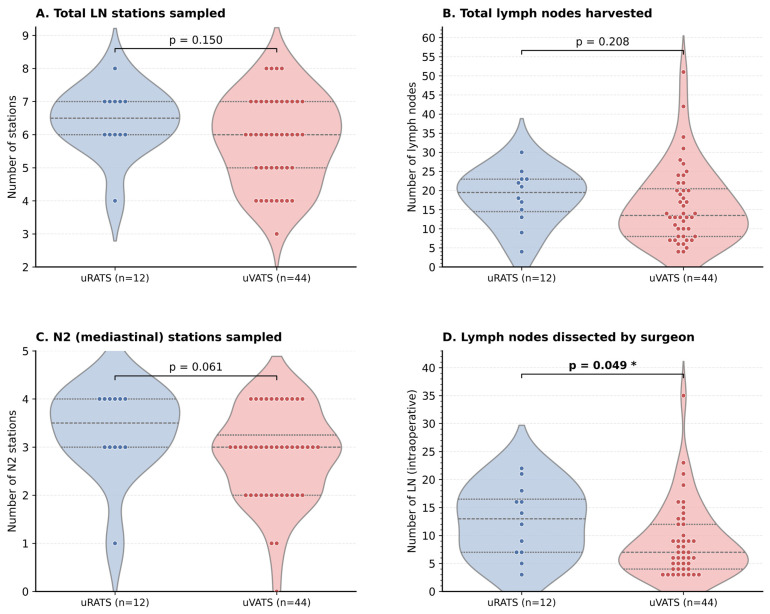
Primary endpoint—lymph-node yield and oncologic radicality. Comparison of four pre-specified lymph-node (LN) dissection metrics between the uRATS (blue; *n* = 12) and uVATS (red; *n* = 44) groups. (**A**) Total number of LN stations sampled, defined as the sum of stations documented intraoperatively by the operating surgeon plus those retrieved from the resection specimen by the pathologist. (**B**) Total number of lymph nodes harvested (intraoperative plus specimen-retrieved). (**C**) Number of mediastinal (N2) stations sampled, out of 7 possible stations (2, 4, 5, 6, 7, 8, 9). (**D**) Number of lymph nodes dissected intraoperatively by the operating surgeon. Filled violins show the kernel density distribution; overlaid dots represent individual patient data points (swarm display). Within each violin, horizontal dashed lines indicate the median (central) and the 25th and 75th percentiles (lower and upper, interquartile range). Between-group comparisons were performed using the Mann–Whitney U test; * *p* < 0.05. Abbreviations: LN, lymph node; N2, mediastinal nodal station; uRATS, uniportal robotic-assisted thoracic surgery; uVATS, uniportal video-assisted thoracoscopic surgery.

Postoperative outcomes are summarized in Table 3 and illustrated in Figure 3, Figure 4 and Figure 5. Routine overnight ICU monitoring, the institutional standard after anatomical lung resection, was utilized for 75.0% of uRATS and 88.6% of uVATS patients (*p* = 0.348); among those admitted, the median ICU length of stay was 12 h, consistent with routine observation rather than complication-driven care. Although the median ICU length of stay was identical (12 h in both groups), the interquartile range was wider in the uRATS arm (1–12 h vs. 12–12 h; *p* = 0.032), reflecting earlier discharge from the ICU in some uRATS patients within the same calendar day. Total pleural drainage volume (median 250 mL vs. 275 mL; *p* = 0.825) did not differ.

Several postoperative recovery metrics favored the uRATS group, particularly air-leak duration, hospital length of stay, and discharge pain. Air-leak duration was markedly shorter after uRATS (median 0 days [IQR 0–1] vs. 2 days [IQR 1–3]; *p* < 0.001; Figure 3A). Eight of 12 uRATS patients (66.7%) experienced no postoperative air leak at all, compared with nine of 44 uVATS patients (20.5%). Prolonged air leak (>5 days) occurred in 0 of 12 uRATS patients versus five of 44 uVATS patients (11.4%; *p* = 0.574). Chest-tube duration was similar between groups (median 2 days in both; *p* = 0.187; Figure 3B). Total hospital length of stay was significantly shorter in the uRATS group (median 2 days [IQR 2–3] vs. 3 days [IQR 3–5]; *p* = 0.022; Figure 3C).

Postoperative pain was consistently lower in the uRATS group. At 12 h postoperatively, the VAS pain score showed a strong numerical trend in favor of uRATS (median 3 [IQR 2–4] vs. 4 [IQR 3–5]; *p* = 0.073; Figure 3D). At hospital discharge, VAS was significantly lower after uRATS (2 [IQR 2–3] vs. 3 [IQR 2–4]; *p* = 0.017; Figure 3E).

In-hospital complication rates were low and similar between groups: any complication, 16.7% vs. 20.5% (*p* = 1.000); minor complication (Clavien–Dindo I–II), 8.3% vs. 11.4% (*p* = 1.000); major complication (Clavien–Dindo ≥ III), 8.3% vs. 9.1% (*p* = 1.000). Thirty-day readmission occurred in 0 of 12 uRATS patients and in two of 44 uVATS patients (4.5%; *p* = 1.000). There was no 30-day mortality in either group. The composite textbook outcome was achieved in 10 of 12 uRATS patients (83.3%) versus 21 of 44 uVATS patients (47.7%; *p* = 0.047; Figure 4), indicating that over a third more uRATS patients completed the full ideal postoperative trajectory.

### 3.4. Pathological Characteristics

Pathological findings are presented in Table 4a. Adenocarcinoma was the predominant histology in both groups (66.7% vs. 77.3%), with the remaining patients having squamous cell carcinoma (8.3% vs. 11.4%) and adenosquamous carcinoma (25.0% vs. 11.4%); the overall histology distribution did not differ between groups (*p* = 0.484). Pathological tumor size (median 3.0 cm [IQR 2.0–4.1] vs. 2.8 cm [IQR 1.5–4.1]; *p* = 0.734) and surgical margin distance (2.0 cm [IQR 1.6–3.0] vs. 2.5 cm [IQR 2.0–4.0]; *p* = 0.284) were comparable. Complete (R0) resection was achieved in every patient in both groups (12/12 [100%] vs. 44/44 [100%]). Pathological T stage (overall *p* = 0.643) and N status (N0 91.7% vs. 77.3%; N1 8.3% vs. 18.2%; N2 0% vs. 4.5%; overall *p* = 0.507) were similarly distributed, as was the simplified pathological stage (stage I 75.0% vs. 59.1%; stage II 16.7% vs. 31.8%; stage III 8.3% vs. 9.1%; overall *p* = 0.564). The rates of lymphatic invasion (66.7% vs. 63.6%; *p* = 1.000), vascular invasion (66.7% vs. 70.5%; *p* = 1.000), perineural invasion (16.7% vs. 4.5%; *p* = 0.198), and visceral pleural invasion (41.7% vs. 56.8%; *p* = 0.515) did not differ between the two groups.

### 3.5. Primary Endpoint—Lymph-Node Yield and Oncologic Radicality

The primary endpoint findings are shown in Table 4b and Figure 5. The operating surgeon dissected a significantly higher number of lymph-node stations (median 5 [IQR 5–5] vs. 4 [IQR 3–5]; *p* = 0.044) and a higher total number of lymph nodes (median 13 [IQR 7–16] vs. 7 [IQR 4–12]; *p* = 0.049; Figure 5D) in the uRATS group than in the uVATS group. After incorporating the additional nodes retrieved from the resection specimen by the pathologist (median 2 stations [IQR 1–2] and 4 nodes [IQR 2–10] in uRATS vs. 2 stations [IQR 1–3] and 6 nodes [IQR 3–10] in uVATS), the total yield-reflecting the combined surgeon-plus-specimen evaluation-showed no statistically significant difference between groups (total stations sampled: median 6 [IQR 6–7] vs. 6 [IQR 5–7]; *p* = 0.150; Figure 5A; total lymph nodes harvested: median 20 [IQR 14–23] vs. 14 [IQR 8–20]; *p* = 0.208; Figure 5B). Thus, the uRATS approach yielded more nodes from the operative field itself, while the final combined yield was numerically higher but not statistically different.

Mediastinal (N2) station sampling showed a strong trend toward more thorough dissection in the uRATS group (median 4 stations [IQR 3–4] vs. 3 [IQR 2–3]; *p* = 0.061; Figure 5C). The ESTS criterion of ≥3 mediastinal stations sampled was fulfilled in 11 of 12 uRATS patients (91.7%) versus 29 of 44 uVATS patients (65.9%; *p* = 0.147). The subcarinal station (station 7) was sampled in 100% of uRATS cases and 88.6% of uVATS cases (*p* = 0.574). An adequate total lymph-node yield (≥10 nodes) was observed in 83.3% of uRATS and 72.7% of uVATS patients (*p* = 0.709). N1 (hilar/intrapulmonary) station sampling was similar between groups (median 3 vs. 3; *p* = 0.636).

Nodal upstaging (conversion from clinical N0 to pathological N1 or N2) was observed in two of 44 uVATS patients (4.5%; both upstaged from cN0 to pN2) and in no uRATS patients (0 of 12; *p* = 1.000). The R0 resection rate—the primary oncologic safety endpoint—was 100% in both arms (12/12 vs. 44/44; *p* = 1.000). Within this cohort, R0 resection was achieved in all patients in both groups, while the directly dissected lymph-node yield was numerically higher in the uRATS arm across several metrics.

### 3.6. Follow-Up and Oncologic Outcomes

At a median follow-up of 12 months (IQR 6–15) in the uRATS group and 19 months (IQR 12–25) in the uVATS group, recurrence had been documented in two of 12 uRATS patients (16.7%) and in six of 44 uVATS patients (13.6%). All-cause mortality during follow-up was recorded in one of 12 uRATS patients (8.3%) and six of 44 uVATS patients (13.6%). The limited and asymmetric follow-up duration (median 7 months longer in the uVATS cohort) precluded meaningful between-group comparison of long-term oncologic endpoints, and no formal Kaplan–Meier survival analysis was therefore conducted; long-term oncologic outcomes will be addressed in future follow-up reports of this cohort.

## 4. Discussion

In this single-center, contemporaneous cohort study of 56 consecutive patients undergoing uniportal minimally invasive anatomical resection for NSCLC, R0 resection was achieved in 100% of patients in both arms, intraoperative lymph-node retrieval by the operating surgeon was numerically higher in the uRATS group, and several perioperative recovery endpoints—including air-leak duration, hospital length of stay, pain at discharge, and the composite textbook outcome—showed unadjusted directional differences in favor of uRATS. These findings, obtained in a thoracic unit already proficient in uniportal thoracoscopy—and therefore in a setting where uVATS represents the contemporary baseline rather than a historical comparator—offer practical information for surgical teams weighing whether to adopt a uniportal robotic program.

Because the present analysis was performed without propensity-score matching or multivariable adjustment, the directional differences described below should be regarded as descriptive observations on a small contemporaneous cohort rather than as adjusted estimates of comparative effectiveness. Our primary-endpoint observations align closely with the broader RATS-versus-VATS literature, notwithstanding the intrinsic differences introduced by the uniportal approach. A reconstructed-patient-data meta-analysis with 51.7 months of median follow-up reported equivalence of overall and disease-free survival between RATS and VATS lobectomy, alongside a modestly higher lymph-node yield and shorter hospital stay with the robotic approach [12]. A prospective-study meta-analysis pooling 614 patients similarly demonstrated more extensive nodal station sampling with RATS (mean difference +1.07 stations, *p* < 0.001) and reduced intraoperative blood loss (mean difference −17.1 mL) [14]. The RVlob randomized controlled trial—the largest to date with 320 patients and mid-term follow-up—established non-inferiority of three-year overall survival for RATS (94.6% vs. 91.5% for VATS) and a small but statistically significant reduction in pain intensity at four weeks postoperatively [12,29]. These long-term comparative-effectiveness data from larger cohorts are summarized here for context only; the present cohort is neither sized nor followed long enough to draw any inference regarding disease-free or overall survival. Within the present uniportal cohort, similar directional patterns were observed across the same domains: more lymph nodes dissected intraoperatively by the surgeon (median 13 vs. 7, *p* = 0.049), a trend toward more thorough mediastinal sampling (≥3 N2 stations in 91.7% of uRATS vs. 65.9% of uVATS), and lower pain at discharge with uRATS. Importantly, the lack of between-group difference in total nodal yield, in the presence of a robust difference in surgeon-dissected nodes, is itself consistent with the methodological framework of our two-compartment reporting strategy: the pathologist-dependent component (N1 retrieval from the en bloc lobectomy specimen) is expected to be similar across surgical approaches because the lobectomy specimen itself is anatomically equivalent, whereas the surgeon-dependent component (separately submitted mediastinal stations) reflects intraoperative dissection quality and may differ between platforms [30]. The convergence of three nodal-quality metrics—surgeon-dissected count, the proportion of patients with ≥3 mediastinal stations sampled (91.7% vs. 65.9%), and the proportion meeting the ≥10-node adequacy threshold (83.3% vs. 72.7%)—collectively, rather than any single metric in isolation, supports the directional pattern observed. A mechanistic interpretation is that the combination of three-dimensional stereoscopic visualization, tremor filtration, and wristed instrument articulation permits more precise dissection around the vascular pedicles and mediastinal fat pad, where thoracoscopic instruments—constrained to rigid shaft trajectories through a single incision—are most likely to under-sample stations 5, 6, 7, and 9. The consistency of these directional effects across a randomized trial, prospective pooled analyses, a propensity-matched uRATS-versus-uVATS series, and the present single-center uniportal cohort is hypothesis-generating, while the narrower primary-operator field of action in uRATS—reflected in our significantly higher surgeon-dissected lymph-node count—may provide a plausible mechanistic anchor [12,14,17,29]. Nevertheless, given the modest sample size and the descriptive nature of these comparisons, the lymph-node-yield findings should be regarded as supporting, rather than as definitively establishing, an oncologic-quality advantage of one approach over the other.

The nodal-upstaging pattern we observed merits specific discussion. In our cohort, nodal upstaging occurred in 0 of 12 uRATS patients and in two of 44 uVATS patients (4.5%), with both upstaged uVATS patients migrating from clinical N0 to pathological N2. Although the between-group difference did not reach statistical significance (*p* = 1.000), the directionality of this finding is noteworthy because existing comparative literature has consistently reported a gradient of upstaging across surgical approaches. A landmark propensity-score-weighted two-center analysis of 911 patients found the highest rate of upstaging with open thoracotomy (21.8%), an intermediate rate with RATS (16.2%), and the lowest rate with VATS (12.3%), with the authors interpreting the VATS finding as reflecting less thorough—rather than less needed—nodal evaluation [31]. A National Cancer Database analysis of propensity-matched robotic and open lobectomies (*n* = 7452 per arm) reported equivalent upstaging rates, supporting the robotic approach as oncologically comparable to open surgery [32]. A single-center European series of 505 patients specifically documented significantly higher mediastinal-station upstaging rates and higher lymph-node counts with RATS compared with VATS [33]. Interpretation of our own data is constrained by the small sample size and by an asymmetric preoperative staging distribution—92% of uRATS patients were clinically N0 versus 82% of uVATS patients—and by the early-stage clinical composition of the uRATS arm overall, such that the absence of upstaging events in the uRATS arm should be regarded as a reflection of these sample-composition characteristics rather than as an indicator of diagnostic equivalence between the two approaches. Nevertheless, the identification of occult N2 disease in two uVATS patients with PET/CT-negative or EBUS-discordant mediastinal nodes underscores the continuing value of thorough intraoperative lymphadenectomy in both approaches, a principle emphasized by contemporary station-based and node-count-based quality standards [31,34].

Several secondary-endpoint signals in our cohort warrant interpretive caution. Operative time was substantially longer with uRATS (median 220 vs. 135 min, *p* < 0.001), a difference that mirrors the early-adoption patterns reported in the broader RATS literature and in uRATS-specific learning-curve studies, in which procedure time has been shown to plateau only after approximately 20 to 30 cases [18,35]. In our 12-case uRATS series, the first six and last six cases demonstrated comparable median console times (202 vs. 192 min), indicating that further case volume will likely be required before institutional-level efficiency gains translate into meaningful operative-time reductions. Postoperative pain at discharge was significantly lower in the uRATS group (*p* = 0.017), a finding that echoes the RVlob randomized trial’s report of a statistically significant—though of limited clinical magnitude—reduction in pain at four weeks postoperatively [29]. We urge caution in over-interpreting the VAS difference given the small robotic sample and the unblinded nature of pain self-reporting in a non-randomized design. The textbook-outcome rate of 83% with uRATS versus 48% with uVATS (*p* = 0.047) is consistent with, though higher than, the approximately 50–70% textbook-outcome range reported in large NSCLC surgical registries [23]. The magnitude of this difference is best understood mechanistically: ‘absence of prolonged air leak’ is a pre-defined component of the textbook-outcome composite, so that air-leak duration directly affects this endpoint; in addition, prolonged air leaks tend to propagate into a longer chest-tube duration and a longer hospital stay, so that a reduction in air-leak duration—as observed in the uRATS arm (median 0 vs. 2 days, *p* < 0.001)—has cascading effects on several downstream components of the composite. Accordingly, the textbook-outcome difference between the two arms should be interpreted cautiously, as a reflection of the dominant air-leak component rather than as an independent indicator of overall surgical quality. Our decision to exclude routine overnight ICU admission from the textbook-outcome composite is conservative and in keeping with published thoracic-surgery textbook-outcome definitions, which likewise do not include ICU admission as a component when such admission reflects institutional standard of care rather than a clinical event [23]. Finally, the baseline pulmonary-function difference favoring the uRATS group (FEV_1_ 102% vs. 85%, *p* = 0.029; FEV_1_/FVC 106% vs. 99%, *p* = 0.048) is acknowledged as a potential confounder of the recovery-endpoint comparison, particularly the favorable air-leak duration and shorter hospital length of stay observed in the uRATS arm—both of which are biologically plausible to be partly mediated by superior baseline pulmonary reserve, since better-preserved lung parenchyma is generally associated with more efficient post-resection re-expansion, fewer prolonged alveolar leaks, and earlier readiness for chest-tube removal and discharge. Mitigating considerations include that all uVATS patients nevertheless met institutional thresholds for anatomical resection, no patient in either group required mechanical ventilation beyond extubation, and the absolute inter-group difference in predicted FEV_1_ remained within the range of routine between-patient variability seen in consecutive thoracic-surgery cohorts. Nevertheless, in the absence of multivariable adjustment, the air-leak and length-of-stay advantages favoring uRATS cannot be fully disentangled from this baseline pulmonary-reserve imbalance and should be interpreted with this caveat in mind.

This study has several limitations that must be explicitly acknowledged. First, the sample size is small—particularly the 12-patient uRATS arm—which limits statistical power, precludes multivariable adjustment for baseline differences, and makes us unable to exclude clinically meaningful effects in endpoints that did not reach statistical significance. This raises a non-trivial risk of Type II error for secondary endpoints that showed numerical trends in favor of uRATS but did not cross the conventional *p* < 0.05 threshold—most notably estimated intraoperative blood loss (median 42 vs. 50 mL, *p* = 0.106), but also VAS pain at 12 h postoperatively (*p* = 0.073) and the proportion of patients with ≥3 mediastinal stations sampled (*p* = 0.061), where a true difference cannot be excluded but the present cohort is underpowered to detect it. For this reason, we have consistently interpreted even statistically significant findings as hypothesis-generating rather than confirmatory. Second, the retrospective, single-center, non-randomized design carries inherent risks of selection bias; although both groups represent consecutive patients from the same contemporaneous period, the choice between uRATS and uVATS was made at the operating team’s discretion on the basis of operating-room scheduling and robotic-platform availability rather than by protocol, which is reflected in the modest baseline pulmonary-function imbalance noted above. Third, formal propensity-score matching was considered but not performed, because the 12-patient uRATS arm is below the cell-size threshold at which propensity-score techniques can be expected to produce stable estimates in a multi-covariate model; instead, baseline characteristics are reported transparently in Table 1, and all clinically relevant differences are directly addressed in the interpretation of findings. Fourth, our initial feasibility experience—comprising the first 10 of these 12 uRATS cases—has been published separately as a surgical-technique case series focused primarily on operative feasibility, with substantially less granular outcome data, no comparator group, and no formal oncologic-endpoint analysis [36]. The present work extends that preliminary report by adding two further uRATS cases, introducing a contemporaneous 44-patient uVATS comparator cohort, expanding outcome capture to primary oncologic-radicality endpoints and the textbook-outcome composite, and performing the first head-to-head comparison of the two uniportal approaches at our institution; no text, tables, or figures have been duplicated from the earlier report. Fifth, follow-up duration is limited and asymmetric between groups (median 12 months for uRATS vs. 19 months for uVATS), precluding meaningful comparison of long-term recurrence and survival endpoints; for this reason, we did not conduct formal Kaplan–Meier analyses, and we frame the reported recurrence and mortality proportions only as early observations. Sixth, cost-effectiveness—a central consideration in the real-world adoption of robotic thoracic surgery, as highlighted in recent evidence syntheses—was not assessed and should be addressed in subsequent work [37]. Seventh, regarding surgeon allocation, all uRATS procedures were performed by a single thoracic surgeon, while the uVATS procedures were performed by a small team of surgeons that included the same individual. This configuration introduces an unavoidable heterogeneity within the uVATS arm; however, it also carries two methodological strengths. Because the same surgeon who performed all uRATS procedures also contributed to the uVATS cohort, the uRATS-versus-uVATS comparison cannot be attributed to surgeon identity alone, and the presence of multiple operators in the uVATS arm arguably renders it a more realistic, real-world comparator than a single-surgeon uVATS series would have been. Nevertheless, a residual surgeon effect cannot be entirely excluded. Larger, multicenter, and ideally randomized comparisons—together with longer follow-up and economic evaluation—will be required to determine whether the differential signals we observed in nodal retrieval, perioperative recovery, and the textbook-outcome composite translate into long-term oncologic and health-economic benefit.

Within these constraints, our findings contribute incremental evidence that, in a thoracic unit experienced in uniportal thoracoscopy, transitioning selected patients to uRATS is technically feasible, associated with comparable short-term oncologic-quality metrics, and shows directional recovery signals that warrant confirmation in larger, multicenter studies. The appropriate framing of our results is therefore not as a demonstration of robotic superiority, but as a single-center signal—coherent with the broader randomized and meta-analytic literature—that warrants confirmation in larger and longer-term comparative studies.

## 5. Conclusions

In this single-center contemporaneous comparison of 56 consecutive patients undergoing uniportal minimally invasive anatomical resection for NSCLC, uRATS showed comparable intraoperative oncologic-quality metrics to uVATS, with a 100% R0 rate in both groups, and was associated with directional differences favoring uRATS in intraoperative lymph-node retrieval by the operating surgeon, postoperative air-leak duration, hospital length of stay, pain at discharge, and the composite textbook outcome. These directional findings were observed at the cost of a substantially longer operative time, consistent with the early phase of the institutional learning curve. Given the small sample size, the asymmetric follow-up duration, and the inherent limitations of a non-randomized retrospective design, our findings should be regarded as a hypothesis-generating signal rather than as definitive evidence of robotic superiority. They support the technical safety and short-term oncologic non-inferiority of the uniportal robotic approach in a thoracoscopy-experienced unit and provide a rationale for larger, multicenter, and ideally randomized comparative studies—including formal cost-effectiveness evaluation and longer-term oncologic follow-up—to confirm whether the perioperative recovery and lymphadenectomy advantages observed here translate into durable clinical and economic benefit.

## Figures and Tables

**Table 1:** Baseline patient, disease, and preoperative characteristics.

Variable	uRATS (*n* = 12)	uVATS (*n* = 44)	*p*-Value
Age (years), median (IQR)	67 (61–74)	68 (62–72)	0.881
Sex, male/female	8/4	34/10	0.470
BMI (kg/m^2^), median (IQR)	27.6 (22.8–29.4)	27.0 (25.0–30.0)	0.749
Ever-smoker, *n* (%)	10 (83.3%)	37 (84.1%)	1.000
Pack-years, median (IQR)	45 (25–62)	45 (19–62)	1.000
FEV_1_ (L), median (IQR)	2.46 (2.15–2.89)	2.29 (1.84–2.69)	0.276
FEV_1_ (% predicted), median (IQR)	102 (91–106)	85 (75–102)	**0.029**
FEV_1_/FVC (%), median (IQR)	106 (104–114)	99 (92–106)	**0.048**
CCI, median (IQR)	5 (3–6)	4 (3–5)	0.264
Age-adjusted CCI, median (IQR)	7 (5–9)	6 (5–8)	0.207
ASA ≥ III, *n* (%)	7 (58.3%)	27 (61.4%)	1.000
ECOG ≥ 1, *n* (%)	7 (58.3%)	30 (68.2%)	0.516
COPD, *n* (%)	5 (41.7%)	16 (36.4%)	0.748
Diabetes mellitus, *n* (%)	5 (41.7%)	11 (25.0%)	0.293
Hypertension, *n* (%)	8 (66.7%)	28 (63.6%)	1.000
Ischemic heart disease, *n* (%)	4 (33.3%)	15 (34.1%)	1.000
Previous/concurrent other malignancy, *n* (%)	3 (25.0%)	8 (18.2%)	0.686
Tumor size on CT (mm), median (IQR)	27 (14–38)	24 (16–35)	0.944
Tumor size on PET-CT (mm), median (IQR)	24 (13–38)	24 (16–33)	0.928
Tumor SUVmax on PET-CT, median (IQR)	7.0 (5.7–10.2)	8.6 (3.1–12.6)	0.920
Neoadjuvant therapy for lung cancer, *n* (%)	0 (0.0%)	1 (2.3%)	1.000

uRATS, uniportal robotic-assisted thoracic surgery; uVATS, uniportal video-assisted thoracoscopic surgery; BMI, body mass index; FEV_1_, forced expiratory volume in one second; FVC, forced vital capacity; CCI, Charlson Comorbidity Index; ASA, American Society of Anesthesiologists physical status classification; ECOG, Eastern Cooperative Oncology Group performance status; COPD, chronic obstructive pulmonary disease; CT, computed tomography; PET-CT, positron emission tomography/CT; SUVmax, maximum standardized uptake value. Continuous variables are expressed as median (interquartile range); categorical variables are expressed as *n* (%). *p*-values are from the Mann–Whitney U test (continuous) or Fisher’s exact test (categorical). Bold values indicate statistical significance (*p* < 0.05).

**Table 2:** Comparison of intraoperative and surgical characteristics between the uRATS and uVATS groups.

Variable	uRATS (*n* = 12)	uVATS (*n* = 44)	*p*-Value
Resection type, *n* (%)			0.548 (overall)
Lobectomy	8 (66.7%)	34 (77.3%)	
Segmentectomy	3 (25.0%)	9 (20.5%)	
Pneumonectomy	1 (8.3%)	1 (2.3%)	
Side (right/left)	9/3	26/18	0.501
Lobe/segment location, *n* (%)			
Upper	7 (58.3%)	27 (61.4%)	
Middle	0 (0.0%)	2 (4.5%)	
Lower	4 (33.3%)	14 (31.8%)	
Left pneumonectomy	0 (0.0%)	1 (2.3%)	
Docking time (min), median (IQR)—uRATS only	5 (5–5)	N/A	—
Console time (min), median (IQR)—uRATS only	190 (179–205)	N/A	—
Operative time (min), median (IQR)	220 (199–240)	135 (124–175)	**<0.001**
Estimated blood loss (mL), median (IQR)	42 (0–58)	50 (48–100)	0.106
Intraoperative RBC transfusion, *n* (%)	0 (0.0%)	0 (0.0%)	1.000
Conversion to open/alternative approach, *n* (%)	0 (0.0%)	0 (0.0%)	1.000
Intraoperative complication, *n* (%)	0 (0.0%)	1 (2.3%)	1.000
Technical difficulty encountered, *n* (%)	2 (16.7%)	2 (4.5%)	0.198

RBC, red blood cell; N/A, not applicable. Docking time and console time are uniquely applicable to the uRATS group. Continuous variables are expressed as median (interquartile range); categorical variables are expressed as *n* (%). Bold values indicate statistical significance (*p* < 0.05).

**Table 3:** Postoperative outcomes, pain, recovery, and textbook outcome.

Variable	uRATS (*n* = 12)	uVATS (*n* = 44)	*p*-Value
Routine postoperative ICU admission, *n* (%)	9 (75.0%)	39 (88.6%)	0.348
ICU stay among admitted (hours), median (IQR)	12 (1–12)	12 (12–12)	**0.032**
Total pleural drainage (mL), median (IQR)	250 (175–288)	275 (150–400)	0.825
Air-leak duration (days), median (IQR)	0 (0–1)	2 (1–3)	**<0.001**
Prolonged air leak (>5 days), *n* (%)	0 (0.0%)	5 (11.4%)	0.574
Chest-tube duration (days), median (IQR)	2 (1–3)	2 (2–4)	0.187
Hospital stay (days), median (IQR)	2 (2–3)	3 (3–5)	**0.022**
VAS at 12 h postop (0–10), median (IQR)	3 (2–4)	4 (3–5)	0.073
VAS at discharge (0–10), median (IQR)	2 (2–3)	3 (2–4)	**0.017**
Any in-hospital complication, *n* (%)	2 (16.7%)	9 (20.5%)	1.000
Minor complication (Clavien I–II), *n* (%)	1 (8.3%)	5 (11.4%)	1.000
Major complication (Clavien ≥ III), *n* (%)	1 (8.3%)	4 (9.1%)	1.000
30-day readmission, *n* (%)	0 (0.0%)	2 (4.5%)	1.000
30-day mortality, *n* (%)	0 (0.0%)	0 (0.0%)	1.000
Textbook outcome, *n* (%)	10 (83.3%)	21 (47.7%)	**0.047**

ICU, intensive care unit; VAS, Visual Analog Scale for pain (0–10). Routine overnight ICU admission is a standard-of-care element at our institution and is not considered a complication. Textbook outcome was defined as the simultaneous achievement of R0 resection, absence of major (Clavien–Dindo ≥ III) complication, absence of prolonged air leak, no 30-day readmission, no 30-day mortality, and ≥6 lymph-node stations sampled. Bold values indicate statistical significance (*p* < 0.05).

**Table 4:** (**a**) Pathological characteristics. (**b**) Primary endpoint—lymph-node yield, station sampling, and oncologic radicality.

**(a)**			
**Variable**	**uRATS (*n* = 12)**	**uVATS (*n* = 44)**	***p*** **-Value**
Histology, *n* (%)			0.484 (overall)
Squamous cell carcinoma	1 (8.3%)	5 (11.4%)	
Adenocarcinoma	8 (66.7%)	34 (77.3%)	
Adenosquamous	3 (25.0%)	5 (11.4%)	
Pathological tumor size (cm), median (IQR)	3.0 (2.0–4.1)	2.8 (1.5–4.1)	0.734
Surgical margin distance (cm), median (IQR)	2.0 (1.6–3.0)	2.5 (2.0–4.0)	0.284
R0 resection, *n* (%)	12 (100.0%)	44 (100.0%)	1.000
Pathological T stage, *n* (%)			0.643 (overall)
T1/T1a/T1b/T1c	5 (41.7%)	18 (40.9%)	
T2a/T2b	5 (41.7%)	18 (40.9%)	
T3/T4	2 (16.7%)	7 (15.9%)	
Tis	0 (0.0%)	1 (2.3%)	
Pathological N status, *n* (%)			0.507 (overall)
N0	11 (91.7%)	34 (77.3%)	
N1	1 (8.3%)	8 (18.2%)	
N2	0 (0.0%)	2 (4.5%)	
Pathological stage (simplified), *n* (%)			0.564 (overall)
I	9 (75.0%)	26 (59.1%)	
II	2 (16.7%)	14 (31.8%)	
III	1 (8.3%)	4 (9.1%)	
Lymphatic invasion, *n* (%)	8 (66.7%)	28 (63.6%)	1.000
Vascular invasion, *n* (%)	8 (66.7%)	31 (70.5%)	1.000
Perineural invasion, *n* (%)	2 (16.7%)	2 (4.5%)	0.198
Visceral pleural invasion, *n* (%)	5 (41.7%)	25 (56.8%)	0.515
**(b)**			
**Variable**	**uRATS (*n* = 12)**	**uVATS (*n* = 44)**	***p*** **-Value**
LN stations dissected by surgeon (*n*), median (IQR)	5 (5–5)	4 (3–5)	**0.044**
LN stations retrieved from specimen (*n*), median (IQR)	2 (1–2)	2 (1–3)	0.281
Total LN stations sampled (*n*), median (IQR) †	6 (6–7)	6 (5–7)	0.150
Lymph nodes dissected by surgeon (*n*), median (IQR)	13 (7–16)	7 (4–12)	**0.049**
Lymph nodes from specimen (*n*), median (IQR)	4 (2–10)	6 (3–10)	0.561
Total lymph nodes harvested (*n*), median (IQR) †	20 (14–23)	14 (8–20)	0.208
N2 (mediastinal) stations sampled, median (IQR)	4 (3–4)	3 (2–3)	0.061
N1 (hilar/intrapulmonary) stations sampled, median (IQR)	3 (3–3)	3 (2–4)	0.636
Subcarinal (station 7) sampled, *n* (%)	12 (100.0%)	39 (88.6%)	0.574
≥3 mediastinal stations sampled (ESTS criterion), *n* (%)	11 (91.7%)	29 (65.9%)	0.147
Adequate LN yield (≥10 nodes), *n* (%)	10 (83.3%)	32 (72.7%)	0.709
Nodal upstaging (cN0 → pN+), *n* (%)	0 (0.0%)	2 (4.5%)	1.000
R0 resection, *n* (%)	12 (100.0%)	44 (100.0%)	1.000

LN, lymph node; ESTS, European Society of Thoracic Surgeons; IQR, interquartile range. † Pre-specified primary endpoint. Total LN stations and total lymph nodes are the sum of nodes dissected intraoperatively by the surgeon and those subsequently retrieved from the resection specimen by the pathologist. Mediastinal (N2) stations are stations 2, 4, 5, 6, 7, 8, and 9; hilar/intrapulmonary (N1) stations are stations 10, 11, 12, 13, and 14. ESTS criterion: ≥3 mediastinal stations, including station 7. Adequate LN yield is defined as ≥10 lymph nodes harvested. Nodal upstaging is defined as conversion from clinical N0 preoperatively to pathological N1 or N2 postoperatively. Bold values indicate statistical significance (*p* < 0.05).

## Data Availability

The de-identified data supporting the findings of this study are available from the corresponding author upon reasonable request. Patient-level data are not publicly available because of institutional ethical and privacy restrictions.
